# Learning Support, Digital Constraints, and Online Learning Outcomes Among Pre-Service Teachers in China’s Government-Funded Teacher Education Program: The Mediating Role of Self-Regulated Learning

**DOI:** 10.3390/bs16081409

**Published:** 2026-08-17

**Authors:** Lanzi Huang, Lingqin Zeng, Jianwen Guo, Xian Liu, Boyuan Duan, Xiaoxuan Zhang, Zhao Cheng

**Affiliations:** 1 School of Education, Hunan First Normal University, Changsha 410205, China; lanzi@hnfnu.edu.cn; 2 Research Center for Innovative Development of Teacher Education in the New Era, Hunan First Normal University, Changsha 410205, China; 3 Graduate School of Education, Peking University, Beijing 100871, China; 4 College of Elementary Education, Capital Normal University, Beijing 100048, China; 7168@cnu.edu.cn; 5 Department of Educational Development & Research, School of Health Professions Education, Faculty of Health, Medicine and Life Sciences, Maastricht University, 6200 MD Maastricht, The Netherlands; xian.liu@maastrichtuniversity.nl; 6 School of Education, Renmin University of China, Beijing 100872, China; 2024000925@ruc.edu.cn; 7 School of Education, Central China Normal University, Wuhan 430079, China; 8 Department of Educational Sciences, Vrije Universiteit Brussel, B-1050 Brussels, Belgium

**Keywords:** perceived learning support, self-regulated learning strategies, smartphone addiction, teacher education, online learning outcomes

## Abstract

Drawing on Self-Determination Theory, this study examines how perceived learning support, smartphone addiction, and user resistance jointly shape online learning outcomes among Chinese pre-service teachers. Data from 1134 participants enrolled in China’s government-funded targeted teacher education program-primarily oriented toward strengthening the supply of teachers for basic education, especially in central and western China, were analyzed using Mplus 8.3. The results showed that perceived learning support was positively associated with learning outcomes, whereas smartphone addiction was negatively associated with them. The role of user resistance was more complex in the full structural model and should therefore be interpreted with caution. Self-regulated learning (SRL) strategies served as a key mediator: perceived learning support improved learning outcomes partly by fostering SRL, while smartphone addiction hindered learning outcomes by weakening SRL. By examining a distinctive cohort of pre-service teachers, this study highlights the importance of supportive learning conditions and institutional context in online learning, and identifies SRL as a key learning process through which learning support and digital constraints are associated with learning outcomes.

## 1. Introduction

Higher education (HE) has undergone a substantial digital transformation in recent years. Specifically, the rapid expansion of online and remote instruction has created an important context for examining the mechanisms that shape learning outcomes and psychological adaptation in digitally mediated environments (Broadbent & Poon, 2015; Hodges et al., 2020; Means et al., 2010). Although the COVID-19 pandemic triggered the large-scale shift to remote teaching, technology has remained a lasting feature of teacher education, making it important to understand the factors associated with successful online learning. Crucially, that unprecedented emergency provided a unique historical window to examine the underlying mechanisms of learning success and psychological adaptation in a fully digital environment, offering valuable insights into today’s ongoing digital integration. For teacher education institutions, understanding these mechanisms is particularly important, as the behavioral patterns developed by students during this period are likely to have long-lasting effects on their future professional practice (Ertmer & Ottenbreit-Leftwich, 2010; Tondeur et al., 2012). This investigation focuses on pre-service teachers, a cohort embodying a distinctive dual identity: they are both the primary participants in digital learning today and the future teachers who will shape technology integration in classrooms. Their ability to achieve online learning success—characterized by achieving intended learning outcomes despite digital challenges—is closely related to their academic and professional readiness. If pre-service teachers struggle with self-regulation or digital distractions during their formative years, such patterns may undermine their future capacity to effectively manage technology-mediated learning environments. Thus, elucidating the psychological pathways that lead to their learning success is essential for the sustainability of the teaching force.

This study was conducted at a renowned normal university in China with a long history in teacher education. The participating university is a major institution for primary teacher education and publicly funded rural teacher preparation in China, making it a distinctive setting for this study. This policy-oriented program is based on targeted recruitment, preparation, and employment, and is intended to strengthen the supply of teachers for basic education, to promote educational equity and rural revitalization (Ministry of Education of the People’s Republic of China, 2021). Reflecting the gender composition of China’s basic education teaching workforce (with an approximately 3:7 male-to-female ratio), this sample offers a unique lens through which to examine how future teachers navigate the tensions between digital support and technological constraints.

To systematically elucidate these dynamics, this study adopts Self-Determination Theory (SDT) as its explanatory framework (Ryan & Deci, 2000, 2017). From an SDT perspective, the fulfillment of basic psychological needs—autonomy, competence, and relatedness—plays a central role in shaping learning motivation and engagement (Ryan & Deci, 2000, 2017). Within this theoretical architecture, perceived learning support is conceptualized as an autonomy-supportive environmental catalyst, whereas self-regulated learning (SRL) strategies serve as the behavioral manifestation of a student’s competence and agency (Zimmerman, 2000; Panadero, 2017). By contrast, smartphone addiction and user resistance are treated as potential constraints that may undermine effective engagement in online learning (Hawi & Samaha, 2016; Kim & Kankanhalli, 2009). By examining the mediating role of SRL strategies, this study aims to map the intricate pathways through which environmental affordances and individual constraints collectively calibrate the learning outcomes of the next generation of teachers.

## 2. Literature Review and Hypotheses Development

### 2.1. Perceived Learning Support and Online Learning Outcomes

Perceived learning support refers to the extent to which students feel that they are receiving support for their learning from their online classes (Wei et al., 2023). It is a multidimensional construct that typically encompasses course design, instructor support, peer collaboration, and learner autonomy (Paechter et al., 2010), and recent studies continue to show that perceived support remains central to students’ engagement and satisfaction in online learning environments (He et al., 2025). In online learning environments, course design plays a particularly important role because it shapes how clearly students understand the structure, expectations, and learning pathways of a course (Martin et al., 2019). A well-designed course is a factor that enhances students’ success in online learning (Jenkins, 2015). In particular, students are more likely to engage with their learning if the instruction makes it clear how to get started and provides them with an introduction to the purpose and structure of the course (Quality Matters, 2023), a pattern that remains consistent in recent research on online engagement and instructional support (Deep et al., 2025). When designing online courses, it is important to consider learners’ diverse needs and learning styles (Gopal et al., 2021). Similarly, Jenkins (2015) underlined how student success can be improved through the development and effective use of course design attributes.

Interaction is another key component of perceived learning support. Learner–instructor interaction or learner–learner interaction is a two-way exchange aimed at exchanging information or ideas relevant to the course content (Alqurashi, 2019). A number of studies have highlighted the essential role of interaction in online courses (Huisman et al., 2018; Kurucay & Inan, 2017). Through interaction, learners may benefit from pedagogical and administrative support, as well as opportunities for knowledge construction, social connections, and communication, thus contributing to knowledge construction, processing, and enhancement, and engaging participants in learning (Hew, 2016).

Perceived learning support is also closely related to learner autonomy. The flexibility offered by online teaching may also empower students to strengthen their learning autonomy, and autonomy-supportive environments provide learners with ample learning opportunities, leading to better individual learning outcomes (Madjar et al., 2013; Margaryan et al., 2015). Recent studies further suggest that autonomy-supportive online environments are closely linked to students’ motivation, engagement, and self-regulated learning in digital contexts (He et al., 2025; Fernández Ortube et al., 2024). In this sense, learner autonomy is a key component of online learning, because it allows individuals to work at their own pace, thus enhancing the individualization of instruction and facilitating active learning. Accordingly, perceived learning support and self-regulated learning jointly shape the learning process and contribute to learners’ perceived learning outcomes (Paechter et al., 2010; Pintrich & De Groot, 1990; Wei et al., 2021). Recent work has further emphasized that supportive online environments can foster learners’ self-regulation, especially in digital contexts that require greater independence in managing tasks, resources, and distractions (Fernández Ortube et al., 2024; Faza & Lestari, 2025).

### 2.2. Smartphone Addiction, User Resistance, and Learning Outcomes

Even in learning contexts, university students and their smartphones have become increasingly intertwined (Judd, 2014). Although smartphones facilitate access to educational resources and collaboration (Chan et al., 2015), research has shown that technology-related distractions are negatively associated with the effort students dedicate to homework and are not conducive to creating a good study environment (Xu, 2015). From an SDT perspective, such technology-induced distractions may function as constraints that interfere with effective engagement in learning. Smartphone addiction (SA) can compete for students’ limited attentional resources, as constant digital stimulation may disrupt concentration and reduce their capacity for self-regulation. Consequently, excessive smartphone use may weaken students’ ability to engage in SRL strategies. University students may be particularly susceptible to smartphone addiction, as demonstrated by a previous study showing a 37.9% prevalence of smartphone addiction in a sample of 4000 Chinese college students (Wang & Zhang, 2015).

Smartphone addiction refers to problematic and difficult-to-control patterns of smartphone use that may interfere with daily functioning and learning (Lin et al., 2014, 2016). The wide range of smartphone applications available to students can become a persistent source of distraction from learning. Smartphone addiction has been found to be not only negatively associated with self-regulation (Ching & Tak, 2017; van Deursen et al., 2015) but also significantly correlated with academic performance (Hawi & Samaha, 2016). More recent studies have reached similar conclusions, suggesting that higher levels of smartphone addiction are associated with lower levels of self-regulation and less favorable learning-related outcomes (Zhang & Wu, 2020). Earlier studies also suggested that time spent on screen-based technologies may be negatively associated with academic performance (Wentworth & Middleton, 2014).

As information and communication technologies (ICTs) continue to evolve, both university lecturers and students are increasingly expected to adapt to new forms of teaching and learning (Barak & Levenberg, 2016a, 2016b; Moore et al., 2011). However, learners do not respond to such changes in the same way (Barak, 2018). For example, Radha et al. (2020) found that students tend to prefer face-to-face instruction to digital learning. Furthermore, learning digitally means that students need to solve problems independently. From an SDT perspective, this oppositional stance may reflect a negative response when learners perceive reduced autonomy in digital learning. When students perceive the transition to digital learning as a mandatory imposition rather than a self-determined choice, they may show lower motivation and weaker engagement.

Such resistance may discourage learners from investing effort into metacognitive monitoring and goal setting, thereby disrupting the behavioral pathways of SRL. Prior research has suggested that stronger resistance to change or to digital learning environments may hinder students’ learning experiences and outcomes (Huang et al., 2012; Rasheed et al., 2020). User resistance (UR) has been one of the primary concerns in IT implementation and adoption because of low levels of motivation to change (Ali et al., 2016). Various factors contribute to students’ reluctance to embrace new technologies, including negative perceptions and skepticism about the effectiveness of technology for learning (Rasheed et al., 2020). Accordingly, resistance to online learning technologies may reduce students’ willingness to engage actively with digital learning environments and may ultimately constrain their learning processes and outcomes.

Perceived learning outcomes refer to the extent to which students believe they have learned effectively from online courses (Kang & Im, 2013). The effective integration of e-learning systems to facilitate teaching and learning is a major ongoing challenge for institutions of higher education. Accordingly, evaluating the quality and effectiveness of online learning has become a key concern for higher education stakeholders (Gress et al., 2010). Perceived learning outcomes have therefore been widely treated as an important indicator of the effectiveness of online learning systems and experiences (Yunusa & Umar, 2021). Previous studies have also asserted that students’ perceived learning outcomes reflect the quality of the education they receive in online settings (Garnjost & Lawter, 2019; Kurucay & Inan, 2017). Research has identified several factors associated with online learning outcomes, including interaction, learning support, learner autonomy, and SRL strategies (Yunusa & Umar, 2021; Artino, 2007). While existing literature has highlighted these individual factors, less attention has been paid to how they may operate together in a unified mediation model. In particular, there remains a need to examine whether SRL strategies help explain how learning support and digital constraints are associated with perceived learning outcomes.

### 2.3. The Mediating Role of Self-Regulated Learning Strategies

Based on Pintrich’s model, self-regulated learning (SRL) involves cognitive, metacognitive, and resource-management strategies that help learners monitor, guide, and regulate their actions to achieve learning goals (Paris & Paris, 2001). SRL strategies typically include elaboration, critical thinking, time management, study environment management, and help-seeking (Pintrich & De Groot, 1990). Individuals with high levels of SRL are able to plan, manage, and regulate their learning processes (Kizilcec et al., 2017). Many studies of online learning environments have shown that high-achieving learners are characterized by high levels of SRL (Jansen et al., 2020), indicating that they are more prone to adopt SRL strategies in their learning (Milligan & Littlejohn, 2016). More recent evidence also suggests that supporting SRL remains a central challenge in technology-supported learning environments, particularly in online and blended settings (Prasse et al., 2024).

One line of research has focused on students’ SRL strategies in online learning, particularly, goal setting, help-seeking, effort regulation, and time management (Milligan & Littlejohn, 2016; Nawrot & Doucet, 2014; Broadbent, 2017). For example, D. Lee et al. (2020) found that time management and metacognitive regulation significantly predicted perceived learning outcomes. Similarly, Jo et al. (2016) showed that time and study management strategies were associated with students’ final grades. Moreover, Cheng and Chau (2013) found that SRL strategies such as elaboration, critical thinking, and metacognition regulation significantly predict e-Portfolio achievement scores. A systematic review by Broadbent and Poon (2015) showed similar results, indicating that SRL strategies (i.e., critical thinking, metacognition, time management, and effort regulation) are positively associated with academic outcomes in online learning.

### 2.4. Research Hypotheses

In the present study, SDT serves as the broader motivational framework for understanding how supportive and constraining learning conditions shape students’ engagement in online learning. Within this framework, perceived learning support is treated as an autonomy-supportive condition that may foster more active engagement and self-regulated learning, whereas smartphone addiction and user resistance are viewed as digital constraints that may undermine effective engagement and self-regulation. SRL, in turn, is conceptualized as a proximal learning process through which these factors may influence perceived learning outcomes. Based on this rationale, the following hypotheses are proposed:

**H_1_.**  *Perceived learning support positively predicts both perceived learning outcomes and the use of SRL strategies.*

**H_2_.**  *The use of SRL strategies is positively associated with perceived learning outcomes.*

**H_3_.**  *Smartphone addiction negatively predicts both perceived learning outcomes and the use of SRL strategies.*

**H_4_.**  *User resistance negatively predicts perceived learning outcomes and the use of SRL strategies, and this relationship is mediated by SRL.*

## 3. Methods

### 3.1. Participants

**Figure 1:**
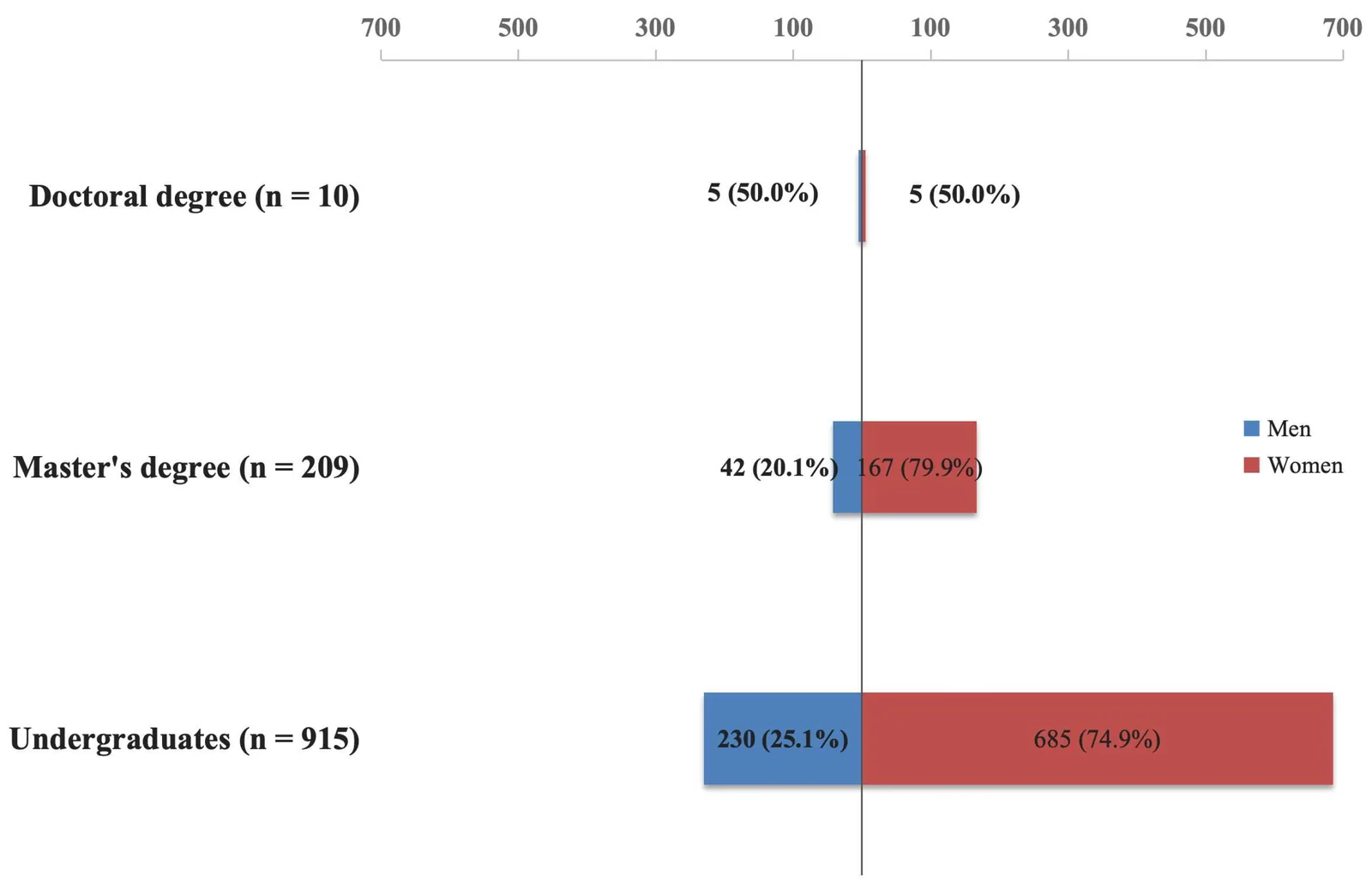
Gender and grade marginal distributions (*N* = 1134).

A total of 1134 pre-service teachers enrolled in China’s government-funded targeted teacher education program participated in this study. The data were collected through an online questionnaire created using Wenjuanxing (WJX, web-based platform; Changsha Ranxing Information Technology Co., Ltd., Changsha, China; https://www.wjx.cn/, accessed on 31 December 2023), a widely used online survey platform in China. The survey link was distributed by teachers and student administrators at the participating university between November and December 2023. This recruitment strategy relied on existing institutional networks within the university to reach students across different academic disciplines and program levels. Institutional coordinators assisted in promoting the study through learning management systems, student forums, and class announcements. A total of 1500 questionnaires were distributed, and after excluding responses with incomplete information, 1134 valid questionnaires were retained for the final analysis, yielding an effective response rate of 75.6%. As shown in Figure 1, the gender ratio of the students was 75.57% female (857) and 24.43% male (277). Of them, 80.7% were undergraduates, 18.4% were master’s degree students, and 0.9% were doctoral students.

**Figure 2:**
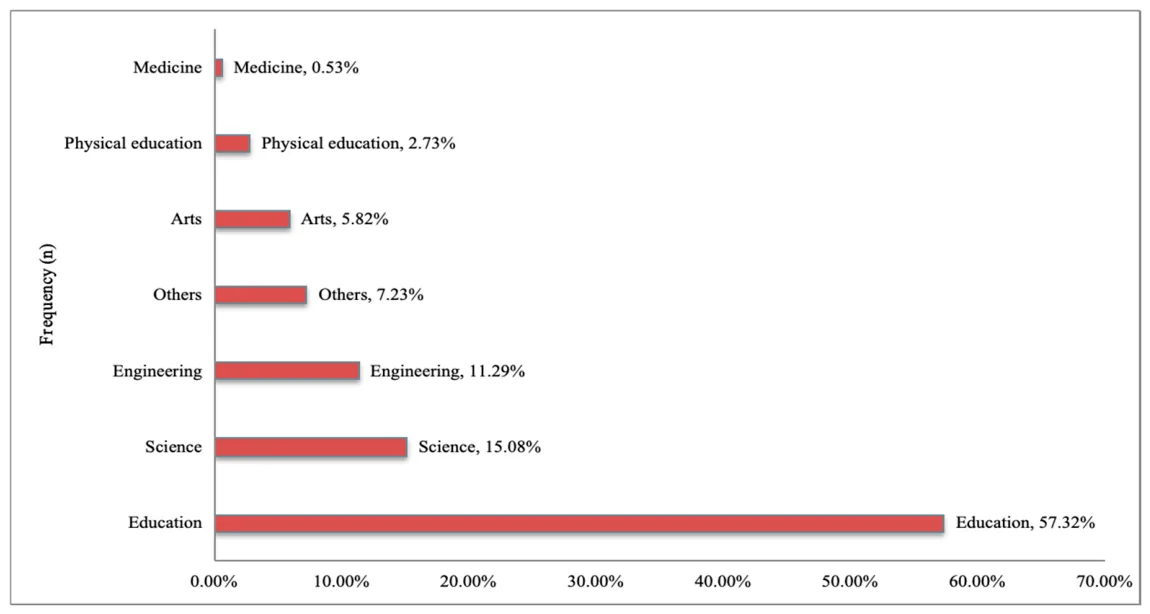
Distribution of participants by discipline.

As shown in Figure 2, the sample exhibited a diverse disciplinary background, with a primary concentration in Education (57.32%), supplemented by science (15.08%) and Engineering (11.29%). Regarding the digital learning infrastructure, a high degree of device redundancy was observed. Table 1 shows that the vast majority of participants utilized smartphones (74.6%) and personal computers (73.3%) as their primary interfaces for online instruction. This technological profile underscores the relevance of examining smartphone-related behavioral constraints within this cohort. In terms of the number of online courses the students were taking, 50.4% took 1–4 online courses, 29.5% took 5–8 online courses, 16.1% took 9–12 online courses, and 4% of students took 13 or more online courses.

In terms of the methods of instruction used in the online classes the respondents took, 61.99% used live streaming, 3.7% used prerecorded video, 2.73% used MOOC platforms, 12.08% used live classes in combination with video classes, 15.34% used live classes in combination with MOOCs, 2.29% used video classes in combination with MOOCs, and 1.85% used other teaching methods. In terms of the online learning platforms used for learning, 22.2% of respondents used DingTalk, 90.9% used Tencent Meeting, 26.6% used the China University MOOC, 62.8% used Super Star Learning, 23.6% used Rain Class, 3.3% used Zoom Meeting, 15.5% used WeChat, 16.8% used Tencent Class, 0.6% used the THEOL online education integrated platform, and 9.2% used other online learning platforms. Separately, when respondents were asked to identify their favorite online learning app, the most frequently selected apps were Super Star Learning (64.2%) and Tencent Class (9.17%), as shown in Table 1.

### 3.2. Measures

College students’ perceived learning outcomes were investigated by measuring perceived learning (Kang & Im, 2013). The measurement consisted of five items, and each item was rated on a 5-point Likert scale ranging from 1 (strongly disagree) to 5 (strongly agree), with higher summed scores indicating more positive perceived learning outcomes. A representative item is: “Using my current mobile devices enables me to accomplish learning tasks more quickly”. This measurement showed good reliability and validity in a previous study of the relationship between e-learners’ self-regulatory efficacy and perceptions of environmental quality in e-learning (J.-K. Lee & Lee, 2008). In the present study, this scale showed acceptable construct validity, with χ^2^ = 52.995, df = 5, *p* < 0.001, RMSEA = 0.092, CFI = 0.990, TLI = 0.948, and SRMR = 0.027; moreover, the scale showed acceptable reliability (Cronbach’s α = 0.89).

Smartphone addiction was measured using the short-form Smartphone Addiction Inventory, a modified version of the 26-item self-reported inventory for assessing the symptoms of smartphone addiction (Lin et al., 2014, 2017). This subscale contains 10 items, with each item rated on a 5-point Likert scale, with higher summed scores indicating that the subject is more addicted to smartphones. A representative item is: “I have substantially increased the amount of time I spend using my smartphone per week over the past three months”. This scale has demonstrated good psychometric properties in Chinese-speaking student samples (Lin et al., 2017). In the present study, this scale showed good construct validity, with χ^2^ = 60.869, df = 31, *p* < 0.01, RMSEA = 0.029, CFI = 0.993, TLI = 0.990, and SRMR = 0.019; moreover, the scale demonstrated good reliability (Cronbach’s α = 0.920).

User resistance was measured using a modified version of the user resistance scale developed by Kim and Kankanhalli (2009). This scale consists of four items, with each item rated on a 5-point Likert scale ranging from 1 (strongly disagree) to 5 (strongly agree), with higher summed scores indicating stronger levels of user resistance. The items include, for example: “I oppose the change from face-to-face to digital learning”. This scale has been shown to have good reliability and validity (Scheel et al., 2022). In the present study, this scale had good construct validity, with χ^2^ = 7.485, df = 2, *p* < 0.050, RMSEA = 0.049, CFI = 0.996, TLI = 0.988, SRMR = 0.008; moreover, the scale demonstrated good reliability (Cronbach’s α = 0.916).

Online SRL strategies were investigated by measuring the use of SRL strategies in the online learning environment (Barnard et al., 2009; Carter et al., 2020). The measurement consisted of five items concerning “goal setting”, four items on “environment structuring,” four concerning “task strategies”, three on “time management”, four concerning “help-seeking”, and four items on “self-evaluation” for a total of 24 items, and each item was rated on a 5-point Likert scale ranging from 1 (strongly disagree) to 5 (strongly agree), with higher summed scores indicating better self-regulation in online learning by students. A representative item is: “Minimizing distraction is an important factor in my choice of location for studying”. This measurement showed good reliability and validity in a previous study of online learning strategies and their effectiveness (Chang et al., 2022). In the present study, this scale had good construct validity, with χ^2^ = 813.519, df = 246, *p* ≤ 0.001; RMSEA = 0.045, CFI = 0.946, TLI = 0.940, and SRMR = 0.040; moreover, the scale showed good reliability (Cronbach’s α = 0.958).

Perceived learning support was investigated using a measurement adapted from the questionnaire on students’ learning expectations and experiences developed by Paechter et al. (2010). The measure consists of four dimensions, with three items about “course design”, five about “interaction with the instructor”, four about “interaction with peer students”, and five about “learner autonomy”, for a total of 17 items. Each item is rated on a 5-point Likert scale ranging from 1 (strongly disagree) to 5 (strongly agree), with higher summed scores indicating better perceived learning support in the online learning environment. A representative item of the measurement is: “Teachers are able to provide feedback on online learning in a timely manner”. This measurement showed good reliability and validity in the present study, with χ^2^ = 317.746, df = 73, *p* ≤ 0.001, RMSEA = 0.054, CFI = 0.952, TLI = 0.940, and SRMR = 0.039; moreover, the scale showed good reliability (Cronbach’s α = 0.935).

### 3.3. Data Analysis

To address the multiple hypotheses proposed in the current study, SPSS 25.0 and Mplus 8.3 were both used for data analysis. First, we analyzed the reliability and validity of all scales. The reliability analysis was mainly conducted using SPSS. The results showed that all of the factors had acceptable reliability coefficients, with Cronbach’s α coefficients ranging from 0.890 to 0.958 (see Table 2). Structural equation modeling (SEM) was performed using Mplus. For confirmatory factor analysis and structural model evaluation, multiple fit indices were employed, including the chi-square statistic (χ^2^), chi-square divided by degrees of freedom (χ^2^/df), the root mean square error of approximation (RMSEA), the Tucker–Lewis index (TLI), the comparative fit index (CFI), and the standardized root mean square residual (SRMR). Following commonly used SEM guidelines, values of CFI and TLI above 0.90 indicate acceptable fit, RMSEA values below 0.08 indicate acceptable fit, and SRMR values below 0.08 indicate acceptable fit (Hu & Bentler, 1999; Schermelleh-Engel et al., 2003). Because the χ^2^ statistic is sensitive to sample size, the χ^2^/df ratio was also reported as a descriptive indicator of model fit.

## 4. Findings

### 4.1. Descriptive Statistics and Correlations

Table 3 presents the descriptive statistics for all study variables. The mean scores ranged from 2.717 to 3.661. Perceived learning support had the highest mean score (M = 3.661, SD = 0.605), followed by online self-regulated learning strategies (M = 3.559, SD = 0.631), perceived learning outcomes (M = 3.290, SD = 0.814), and smartphone addiction (M = 3.282, SD = 0.774). User resistance showed the lowest mean score (M = 2.717, SD = 0.907). All five scales demonstrated good reliability. As shown in Table 3, perceived learning outcomes were positively associated with perceived learning support and self-regulated learning, but negatively associated with smartphone addiction and user resistance. In addition, perceived learning support was strongly positively related to self-regulated learning, whereas smartphone addiction was positively related to user resistance.

### 4.2. The Mediating Analysis

#### 4.2.1. Single-Predictor Mediation Models

We first estimated three single-predictor mediation models using bootstrapping in PROCESS for SPSS, with SRL as the mediator between each predictor and PLO. All indirect effects were significant because the 95% bootstrap confidence intervals did not include zero. PLS had a positive indirect effect on PLO through SRL (β = 0.318, SE = 0.049, 95% CI [0.224, 0.412]). In contrast, SA (β = −0.077, SE = 0.025, 95% CI [−0.125, −0.028]) and UR (β = −0.050, SE = 0.021, 95% CI [−0.092, −0.010]) showed significant negative indirect effects through SRL. These results show that, when examined separately, higher PLS was associated with better perceived learning outcomes through greater use of SRL strategies, whereas higher SA and UR were associated with poorer outcomes through reduced SRL.

#### 4.2.2. Full Structural Equation Model

**Figure 3:**
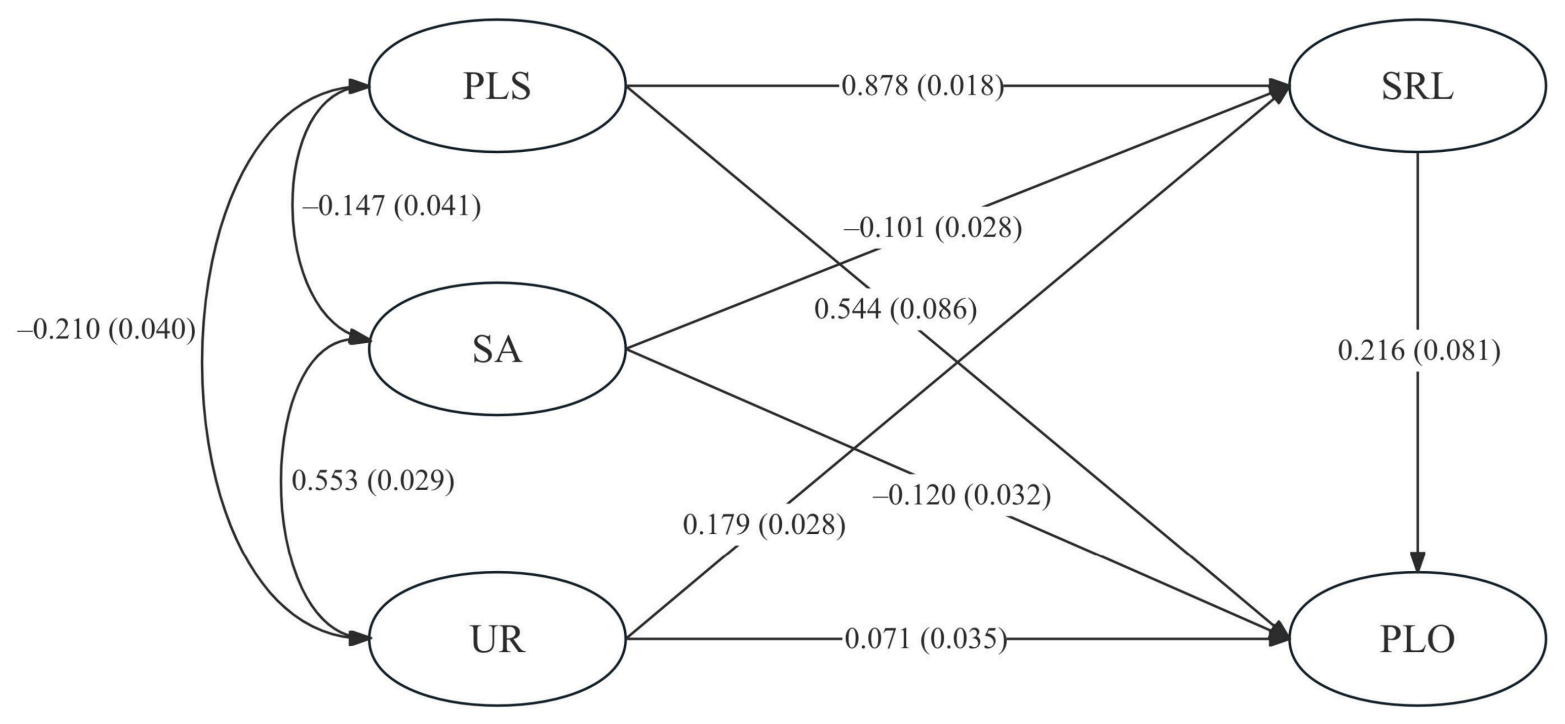
Mediation effects through SRL (*N* = 1134).

We then estimated a full structural equation model in which PLS, SA, and UR were entered simultaneously (Figure 3). The model showed acceptable fit to the data on balance: χ^2^ = 1380.761, df = 199, χ^2^/df = 6.94, *p* < 0.001, RMSEA = 0.072, CFI = 0.938, TLI = 0.928, and SRMR = 0.051 (Hu & Bentler, 1999; Schermelleh-Engel et al., 2003). Although the χ^2^/df value was higher than commonly recommended levels, the RMSEA, CFI, TLI, and SRMR values were within acceptable ranges, suggesting that the model fit was acceptable on balance. In this model, all estimated paths reached statistical significance, although the directions of the UR paths differed from the hypothesized negative direction in the full structural model. PLS positively predicted both SRL (β = 0.878, *p* < 0.001) and PLO (β = 0.544, *p* < 0.001), whereas SA negatively predicted SRL (β = −0.101, *p* < 0.001) and PLO (β = −0.120, *p* < 0.001). SRL also positively predicted PLO (β = 0.216, *p* = 0.007).

Notably, after controlling for PLS and SA, UR showed positive associations with SRL (β = 0.179, *p* < 0.001) and PLO (β = 0.071, *p* = 0.044), which differed from the negative effects observed in the single-predictor model. This pattern suggests a possible suppression effect. Because UR was negatively correlated with PLS and positively correlated with SA, its negative zero-order association with PLO may partly reflect shared variance with lower perceived learning support and higher smartphone addiction. Once these shared components were controlled, the UR-specific association became positive.

As shown in Table 4, the bootstrapped indirect effects in the full structural model further supported the mediating role of SRL. The indirect effect of PLS on PLO through SRL was significant (β = 0.190, SE = 0.070, *p* = 0.007, 95% CI [0.054, 0.329]). The indirect effect of SA was also significant and negative (β = −0.022, SE = 0.011, *p* = 0.037, 95% CI [−0.050, −0.006]). In contrast to the single-predictor model, UR showed a significant positive indirect effect through SRL (β = 0.039, SE = 0.015, *p* = 0.010, 95% CI [0.013, 0.072]), consistent with the suppression pattern identified above.

## 5. Discussion

The results show that perceived learning support and self-regulated learning (SRL) were positively associated with perceived learning outcomes, whereas smartphone addiction was negatively associated with both SRL and perceived learning outcomes. These findings are broadly consistent with Self-Determination Theory (SDT), in which supportive learning conditions facilitate more effective engagement, while digital constraints may undermine students’ ability to manage their learning processes. In the present study, perceived learning support can be understood as an autonomy-supportive resource that helps sustain students’ engagement in online learning, which is in line with prior research highlighting the importance of environmental support for perceived learning effectiveness (e.g., Baber, 2020). At the same time, the negative association between smartphone addiction and online learning outcomes is consistent with earlier studies showing that problematic smartphone use may interfere with self-regulation and academic functioning (e.g., Hawi & Samaha, 2016). However, the role of user resistance was more complex. Although it showed negative associations in the single-predictor model, its direct and indirect effects became positive in the full structural model after controlling for perceived learning support and smartphone addiction, suggesting a possible suppression effect that should be interpreted with caution.

The positive association between perceived learning support and SRL is consistent with the SDT perspective that supportive learning environments can foster more active and self-directed forms of engagement. For pre-service teachers in this study, a well-structured online course, timely instructor feedback, and supportive interaction may strengthen learners’ sense of competence and relatedness, thereby encouraging them to regulate their learning more actively. This interpretation is consistent with Ryan and Deci’s (2017) argument that students are more likely to internalize learning goals and mobilize cognitive and metacognitive resources when the learning environment supports their basic psychological needs.

The mediation analyses further showed that SRL played an important role in explaining how perceived learning support and smartphone addiction were associated with perceived learning outcomes. In the single-predictor mediation models, higher perceived learning support was associated with better outcomes through stronger SRL, whereas higher smartphone addiction and user resistance were associated with poorer outcomes through weaker SRL. In the full structural model, the indirect effects of perceived learning support and smartphone addiction through SRL remained significant, which suggests that SRL is an important learning process linking supportive and constraining conditions to students’ perceived learning outcomes. In this respect, the present findings extend earlier work that emphasized direct associations between online learning experiences and perceived learning outcomes (e.g., Haverila, 2011) by showing that SRL also functions as an important intervening process.

One possible explanation for the negative association between smartphone addiction and SRL is that smartphone-related interruptions may disrupt several concrete components of self-regulated learning. In online learning contexts, frequent smartphone use may fragment study time, weaken attentional control, reduce learners’ ability to structure a focused study environment, and undermine sustained goal-directed engagement. These mechanisms may help explain why higher levels of smartphone addiction were associated with weaker SRL and poorer perceived learning outcomes in the present study. In this sense, the findings are broadly consistent with previous research showing that problematic smartphone use is related to poorer self-regulation and less favorable academic outcomes.

By contrast, the findings for user resistance were more complicated than originally expected. In the single-predictor model, higher user resistance was associated with poorer outcomes through weaker SRL. However, in the full structural model, both the direct and indirect effects of user resistance became positive after controlling for perceived learning support and smartphone addiction. This pattern may reflect a suppression effect or shared variance among the predictors. Therefore, the role of user resistance in the present study should not be interpreted as a straightforward negative predictor. Instead, it appears to be contingent on the broader configuration of supportive and constraining factors in the model, and this issue requires further examination in future research.

## 6. Conclusions

This study examined how perceived learning support (PLS), smartphone addiction (SA), and user resistance (UR) relate to perceived learning outcomes through self-regulated learning (SRL) among pre-service teachers at a major Chinese normal university with a long tradition in teacher education. The findings show that perceived learning support was positively associated with both SRL and perceived learning outcomes, whereas smartphone addiction was negatively associated with both. SRL also played an important mediating role in explaining how supportive and constraining factors were linked to online learning outcomes. At the same time, the role of user resistance appeared to be more complex in the full structural model and should therefore be interpreted with caution.

The study contributes to the literature by bringing together supportive and constraining factors within a single analytical framework and by highlighting SRL as a key learning process in online teacher education. From a practical perspective, the findings suggest that teacher education institutions should strengthen autonomy-supportive instructional environments, provide timely feedback and meaningful interaction, and explicitly foster students’ self-regulated learning capacities. In particular, given the unique mission of China’s government-funded teacher education program, universities should treat digital self-regulation as an important professional competency and integrate it more systematically into coursework, online learning activities, and teaching practica. By doing so, teacher education programs may better prepare future teachers to cope with digital distractions, engage more effectively with technology-supported learning and adapt to increasingly digital teaching environments.

## 7. Limitations and Future Prospects

This study had several limitations. First, this study utilized a cross-sectional design, which limits the ability to draw definitive causal inferences. While the structural paths were grounded in robust theoretical frameworks (e.g., SDT), we acknowledge that the cross-sectional nature might lead to biased estimates of mediating effects (Fu et al., 2022). Second, this study focused on perceived learning outcomes rather than objective indicators such as examination scores or course grades. Although perceived learning outcomes are widely used in online learning research and capture students’ subjective evaluations of their learning experience, they may not fully reflect actual academic performance.

Future research could address these limitations in several ways. Longitudinal or cross-lagged panel designs could further validate the temporal precedence and causal directions among perceived support, SRL, and online success. Future research could combine self-reported measures with objective indicators to provide a more comprehensive assessment of learning outcomes. In addition, although our sample focuses on pre-service teachers—a critical cohort for future education—the results may not be directly generalizable to students in primary or secondary vocational schools. Future studies should explore whether these psychological mechanisms remain invariant across diverse educational stages and socio-economic backgrounds. Finally, this study treated SRL strategies as a composite construct without examining the specific contributions of its individual dimensions (e.g., goal setting, environment structuring, and help-seeking). Because different regulatory components may interact differently with smartphone addiction and user resistance, future research should adopt a more fine-grained approach to identify which SRL dimensions are most sensitive to digital constraints and which may serve as stronger protective factors in online learning.

## Figures and Tables

**Table 1:** Descriptive Statistics of Approaches and Equipment of Online Learning.

		*N*	%			*N*	%
Most popular way	Live class	703	61.99	Favorite app	Ding Talk	66	5.8
	Video class	42	3.7		Super Star Learning	728	64.2
	MOOC	31	2.73		MOOC	81	7.14
	Live class + Videos class	137	12.08		Tencent Class	104	9.17
	Live class +MOOC	174	15.34		Rain Class	28	2.47
	Videos class + MOOC	26	2.29		Zoom	5	0.44
	Others	21	1.85		WeChat	23	2.03
	Total	1134	100		Tencent Meeting	30	2.65
Device	Phone	846	74.60		THEOL	1	0.09
	Computer	831	73.30		Others	68	6
	Tablet	313	27.60		-	-	-
	No equipment	8	0.70		-	-	-
	Total	1998	176.20		Total	1134	100

**Table 2:** Validity and Reliability Evidence.

Construct	Dimensions	AVE	Construct Reliability (CR)	Cronbach’s α
Perceived learning support	Course design	0.623	0.832	0.935
	Interaction with instructors	0.485	0.789	
	Interaction with peer students	0.585	0.849	
	Learner autonomy	0.648	0.846	
Perceived learning outcomes	-	0.624	0.891	0.890
Online Self-regulated Learning Strategies	Goal setting	0.652	0.903	0.958
	Environment structuring	0.569	0.841	
	Task strategies	0.576	0.844	
	Time management	0.571	0.799	
	Help seeking	0.517	0.809	
	Self-evaluation	0.637	0.840	
Smartphone addiction	Compulsive behavior	0.583	0.807	0.920
	Functional impairment	0.625	0.833	
	Withdrawal	0.755	0.861	
	Tolerance	0.729	0.843	
User resistance	-	0.724	0.912	0.916

**Table 3:** Pearson’s Correlations for the Variables Measured.

No.	Variable	1	2	3	4	5
1	PLO	-				
2	PLS	0.665 **	-			
3	SA	−0.151 **	−0.119 **	-		
4	SRL	0.637 **	0.778 **	−0.117 **	-	
5	UR	−0.154 **	−0.205 **	0.535 **	−0.088 **	-
	M	3.290	3.661	3.282	3.559	2.717
	SD	0.814	0.605	0.774	0.631	0.907

Note. PLO = perceived learning outcomes; PLS = perceived learning support; SA = smartphone addiction; SRL = self-regulated learning; UR = user resistance; M = mean; SD = standard deviation. ** *p* < 0.01.

**Table 4:** Percentile bootstrap confidence intervals of the indirect effects (*N* = 1134).

Mediation Path	Effect	β	SE	*p*	Boot LLCI	Boot ULCI
PLS → SRL → PLO	Total effect	0.734	0.026	<0.001	0.675	0.779
	Indirect	0.190	0.070	0.007	0.054	0.329
	Direct	0.544	0.086	<0.001	0.368	0.703
UR → SRL → PLO	Total effect	0.110	0.033	0.001	0.047	0.176
	Indirect	0.039	0.015	0.010	0.013	0.072
	Direct	0.071	0.035	0.044	0.003	0.142
SA → SRL → PLO	Total effect	−0.142	0.034	<0.001	−0.210	−0.077
	Indirect	−0.022	0.011	0.037	−0.050	−0.006
	Direct	−0.120	0.032	<0.001	−0.183	−0.055

## Data Availability

The original contributions presented in the study are included in the article; further inquiries can be directed to the corresponding author.
